# Improving manual oxygen titration in preterm infants by training and guideline implementation

**DOI:** 10.1007/s00431-016-2811-x

**Published:** 2016-11-26

**Authors:** Henriëtte A. van Zanten, Steffen C. Pauws, Evelien C. Beks, Ben J. Stenson, Enrico Lopriore, Arjan B. te Pas

**Affiliations:** 1 Division of Neonatology, Department of Pediatrics, Leiden University Medical Center, J6-S, PO Box 9600, 2300 RC Leiden, The Netherlands; 2 TiCC, Tilburg University, Tilburg, The Netherlands; 3 Neonatal Unit, Simpson Centre for Reproductive Health, Royal Infirmary of Edinburgh, Edinburgh, UK

**Keywords:** Preterm infant, Targeting oxygen, Apnoea, Hypoxaemia, Hyperoxaemia

## Abstract

To study oxygen saturation (SpO_2_) targeting before and after training and guideline implementation of manual oxygen titration, two cohorts of preterm infants <30 weeks of gestation needing respiratory support and oxygen therapy were compared. The percentage of the time spent with SpO_2_ within the target range (85–95%) was calculated (%SpO_2_-wtr). SpO_2_ was collected every minute when oxygen is >21%. ABCs where oxygen therapy was given were identified and analyzed. After training and guideline implementation the %SpO_2_-wtr increased (median interquartile range (IQR)) 48.0 (19.6–63.9) % vs 61.9 (48.5–72.3) %; *p* < 0.005, with a decrease in the %SpO_2_ > 95% (44.0 (27.8–66.2) % vs 30.8 (22.6–44.5) %; *p* < 0.05). There was no effect on the %SpO_2_ < 85% (5.9 (2.8–7.9) % vs 6.2 (2.5–8) %; ns) and %SpO_2_ < 80% (1.9 (1.0–3.0) % vs 1.7 (0.8–2.6) %; ns). In total, 186 ABCs with oxygen therapy before and 168 ABCs after training and guideline implementation occurred. The duration of SpO_2_ < 80% reduced (2 (1–2) vs 1 (1–2) minutes; *p* < 0.05), the occurrence of SpO_2_ > 95% did not decrease (73% vs 64%; ns) but lasted shorter (2 (0–7) vs 1 (1–3) minute; *p* < 0.004).

*Conclusion*: Training and guideline implementation in manual oxygen titration improved SpO_2_ targeting in preterm infants with more time spent within the target range and less frequent hyperoxaemia. The durations of hypoxaemia and hyperoxaemia during ABCs were shorter.

**Table:** 

**What** **is Known:** • *Oxygen saturation targeting in preterm infants can be challenging and the compliance is low when oxygen is titrated manually.* • *Hyperoxaemia often occurs after oxygen therapy for oxygen desaturation during apnoeas.*
**What is New:** • *Training and implementing guidelines improved oxygen saturation targeting and reduced hyperoxaemia.* • *Training and implementing guidelines improved manual oxygen titration during ABC.*

## Introduction

Oxygen is the most commonly used therapy in neonatal intensive care units (NICUs) [34]. To assure adequate delivery of oxygen to the tissue without creating oxygen toxicity [29], infants admitted to the NICU are continuously monitored using pulse oximetry. Oxygen is titrated manually to maintain the pulse oxygen saturation (SpO_2_) within target ranges, but this can be challenging. Several studies reported low compliance in oxygen saturation targeting and described a tendency of caregivers to accept higher SpO_2_ [3, 9, 20–22, 26, 33]. It has been suggested that caregivers are more focused to prevent hypoxaemia rather than hyperoxaemia [4, 31]. However, improving the knowledge of caregivers in the hazards of hyperoxaemia could lead to more vigilance for alarm settings and oxygen titration and thus decrease the time outside target ranges in preterm infants considerably [4].

Oxygen is most frequently manually titrated when an apnoea occurs, defined as a respiratory pause >20 s and/or shorter accompanied by bradycardia or cyanosis, hypotonia, and pallor (usually termed ABC: apnoea, bradycardia, cyanosis) [12]. We recently demonstrated that manual titration of oxygen therapy in preterm infants during ABC unintendedly led to the occurrence of hyperoxaemia (SpO_2_ > 95%) [33]. To improve the compliance, especially during ABCs, all neonatal caregivers in our NICU received an additional training about the risk for hypoxaemia and hyperoxaemia, and a guideline for manual oxygen titration was introduced.

Efforts have been taken to increase the nurses’ compliance in SpO_2_ targeting by creating awareness by training and implementing guidelines, with variable success [2, 11, 13, 14, 18, 19]. We aimed to investigate the effect of training combined with an oxygen titration guideline on the proportion of time SpO_2_ was within target range (%SpO_2_-wtr) and the occurrence and duration of hypoxaemia and hyperoxaemia during and after ABCs.

## Methods

A prospective observational study was performed in the NICU of the Leiden University Medical Center (LUMC), which is a tertiary level perinatal center in the Netherlands with an average of 650 intensive care admissions per year. This study was an audit and part of a quality improvement project and did not need to comply with the Dutch law on Medical Research in Humans; the Research Ethics Committee issued a statement of no objection. All infants <30 weeks of gestation admitted to the NICU in LUMC between March 2013 and December 2013 (before training and guideline) and between February 2014 and November 2014 (after training and guideline) were retrospectively compared.

**Fig. 1:**
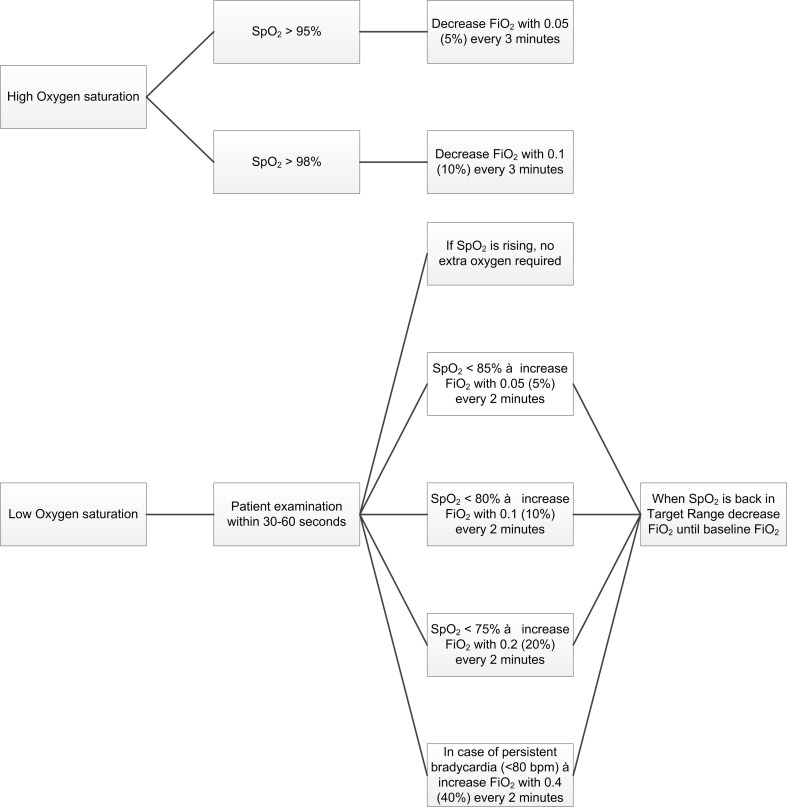
Oxygen titration guideline

To increase awareness in SpO_2_ targeting and oxygen titration, all caregivers were trained in a months’ period (January 2014). Before the afternoon shift started, nurses were asked to attend a lesson that lasted 30–45 min. Each session was attended by 6–8 nurses. An attendance list was updated to make sure every nurse attended the lesson. The medical staff was trained separately during a grand round session. The training was given by the nurse (first author) or the neonatal consultant (last author) responsible for the quality improvement project. During this training the results of our previous study was discussed, which demonstrated frequent occurrence of hyperoxaemia after ABCs where oxygen therapy was given [33]. Caregivers were also educated about the risks for preterm infants exposed to frequent hypoxaemia and hyperoxaemia. To pursue a uniform approach for oxygen titration, a guideline for oxygen titration was introduced and discussed (Fig. 1). After the training, the nurse and the consultant responsible for the project were available during the daytime and frequently actively approached the staff whether there were questions or issues related to the oxygen titration and/or the guideline. Also, the medical staff was asked to standardly check the oxygen saturation distribution during the daily rounds.

The guideline was specially developed for a randomized trial comparing manual versus automated oxygen titration [32]. During the trial, the nurses used the guideline during the manual periods. The guideline was then discussed by members of the project and the nurses who received special training in ventilation. Based on their feedback, small amendments were made to make it more practicable for the nurses.

All preterm infants receiving respiratory support (endotracheal and noninvasive ventilation) in the NICU were included in the study. Infants with major congenital heart disease with different oxygen saturation target ranges were excluded. All infants received routinely a loading dose of 10 mg/kg caffeine directly after birth followed by 5 mg/kg/day. Dopram (2 mg/kg/h) was given in case of refractory apnoeas. Respiratory support was given by a mechanical ventilator (AVEA, Carefusion, Houten, The Netherlands), which is connected to the patient data management system (PDMS) (Metavision; IMDsoft, Tel Aviv, Israel). SpO_2_ was measured using Masimo SET Radical pulse oximeter (software version 46.02) (Masimo Radical, Masimo Corporation, Irvine CA, USA), integrated into the bedside monitor (Philips Healthcare Nederland, Eindhoven, The Netherlands). The pulse oximeter probe was placed around the hand or foot of the infant (right hand in case of a patent ductus arteriosus). Basic characteristics were collected from the patients’ files in PDMS. All clinical parameters were collected every minute from PDMS. In both periods, the SpO_2_ target range (TR) was 85–95% when FiO_2_ > 0.21, and the alarm limits were set at 84 and 96%. Before the start of each shift, the TR and alarm setting were checked by the nurse.

%SpO_2_-wtr, SpO_2_ < 85%, and SpO_2_ > 95%, when FiO_2_ > 0.21 was calculated for each patient during the time period, infants were respiratory supported. Additionally, all ABC events were documented and evaluated in all preterm infants on noninvasive ventilation (nasal CPAP and noninvasive intermittent mandatory ventilation). ABC was defined as apnoea (>20 s or shorter), accompanied with bradycardia (<80 beats per minute (bpm)) and cyanosis (SpO_2_ < 80%). Every ABC was evaluated in detail by documenting the following characteristics: depth and duration of bradycardia, depth and duration of SpO_2_ < 80%, baseline FiO_2_, additional FiO_2_, the duration of the additional FiO_2_, and incidence and duration of SpO_2_ > 95%. Hypoxaemia was defined as SpO_2_ < 80% and hyperoxaemia as SpO_2_ > 95%.

All ABCs were manually identified in PDMS and analyzed starting from the occurrence of an ABC until the additional oxygen given returned to the baseline oxygen that was given before the ABC occurred.

## Statistical analyses

Quantitative data presented as median interquartile range (IQR), mean (SD), or number (percentage) were appropriate. Time with SpO_2_ within various ranges for FiO_2_ > 21% were collated for each infant individually before and after training and aggregated as proportions of the recorded time (median and IQR). Statistical analysis comprised nonparametric Kruskal-Wallis rank sum test. The Mann-Whitney U test for nonparametric comparisons for continuous variables is used to compare the patients’ characteristics and the ABC characteristics. *P* values < 0.05 were considered to indicate statistical significance. Statistical analyses were performed using IBM SPSS Statistics version 23 (IBM Software, NY, USA, 2012) and R 3.2.0 (R Core Team (2015). R: A language and environment for statistical computing. R: A foundation for Statistical Computing, Vienna, Austria. URL https://www.R-project.org/).

We considered an increase of 10% SpO_2_-wtr clinically relevant. In previous studies, the standard deviation of the mean %SpO_2_-wtr was 16 [32]. To detect a change of 10% SpO_2_-wtr in each period by a Kruskal-Wallis test with an 80% power with a significant level of 0.05 (two-tailed test), at least 44 patients of each group were required. We calculated this by running a simulation taking samples from two normal distributions with means 0 and 10 and a standard deviation of 16 to model the clinically relevant increase in %SpO2-wtr.

## Results

### Patient characteristics

During two study periods of 10 months, in total 136 infants <30 weeks of gestation were admitted to our NICU, of which 79 infants before and 57 infants after education and guideline for oxygen titration. The median IQR gestational age was (28 + 2 (27 + 3–29) vs 28 + 3 (26 + 4–29) weeks; ns) and birthweight (1090 (857–1277) vs 1000 (855–1206); ns) were not different (Table 1).

**Table 1.** Patient characteristics

	Before training *N* = 79	After training *N* = 57	*p* value
Gestational age at birth (weeks), median (IQR)	28 + 2 (27 + 3–29)	28 + 3 (26 + 4–29)	0.36^a^
Birthweight (grams) median (IQR)	1090 (857–1277)	1000 (855–1206)	0.56^a^
Male sex, no. (%)	46 (58)	32 (56)	0.96^b^
Caesarean delivery, no. (%)	39 (49)	31 (54)	0.56^b^
Singletons, no. (%)	51 (65)	39 (68)	0.26^b^
Apgar at 5 min median, (IQR)	7 (7–8)	7 (6–9)	0.66^a^
Days on respiratory support, median (IQR)	9 (3–14)	8 (4–24)	0.89^a^
Length of stay on NICU, median (IQR)	15 (8–25)	19 (8–35)	0.32^a^

^a^Independent samples Mann-Whitney U test

^b^Chi-square test

### Effect of training and guideline on the %SpO_2_-wtr

**Fig. 2:**
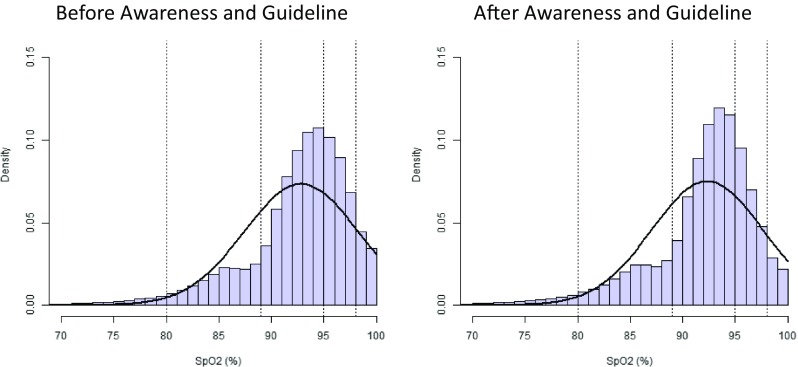
Time with SpO_2_ within various ranges collated over all infants and aggregated as a total proportion of the recorded time. The *smoothed bell-shaped line* represents a fitted normal density function parameterized by the empirical mean and standard deviation estimated from the proportion data of the recorded time within various SpO_2_ ranges. The distribution of the proportional recorded time data is slightly negatively skewed with a *long tail at the left* and a *higher mass at the right-hand side*, when compared with a normal distribution

There was a small but significant decrease median SpO_2_, where IQR remained similar (before vs after training: 94 (91–96) % vs 93 (91–96) %; *p* = 0.02). After training and guideline implementation, the %SpO_2_-wtr significantly increased (before vs after training: 48.0 (19.6–63.9) % vs 61.9 (48.5–72.3) %; *p* < 0.005), with a concomitant decrease in SpO_2_ > 95% (44.0 (27.8–66.2) % vs 30.8 (22.6–44.5) %; *p* < 0.05) and a nonsignificant decrease in SpO_2_ > 98% (9.4 (4.2–26.8) % vs 6.1 (2.3–12.1) %; ns). %SpO_2_ < 85% remained similar (5.9 (2.8–7.9) % vs 6.2 (2.5–8.0) %; ns) as well as for SpO_2_ < 80% (1.9 (1.0–3.0) % vs 1.7 (0.8–2.6) %; ns) (Table 2) (Fig. 2).

**Table 2.** Median (IQR) in different saturation ranges

	Before training	After training	*p* value^a^
%SpO_2_ < 80%	1.9 (1.0–3.0)	1.7 (0.8–2.6)	ns
%SpO_2_ < 85%	5.9 (2.8–7.9)	6.2 (2.5–8.0)	ns
%SpO_2_ − wtr 85–95%	48.0 (19.6–63.9)	61.9 (48.5–72.3)	<0.005
%SpO_2_ > 95%	44.0 (27.8–66.2)	30.8 (22.6–44.5)	<0.05
%SpO_2_ > 98%	9.4 (4.2–26.8)	6.1 (2.3–12.1)	0.06

^a^Time with SpO_2_ within various ranges collated for each infant individually and aggregated as proportions of the recorded time median (IQR). Statistical analysis comprised nonparametric Kruskal-Wallis rank sum test

### Effect of training and guideline on the occurrence of ABCs

Before training and guideline implementation, 79 infants received noninvasive respiratory support, of which 29/79 infants had a total of 186 ABCs where extra FiO_2_ was given. After training and guideline implementation, 57 infants received noninvasive respiratory support, and 28/57 had a total of 168 ABCs (Table 3). After training and guideline implementation, the depth and duration of bradycardia did not change. Although no difference was observed in the depth of SpO_2_ < 80% during ABC, the duration of SpO_2_ < 80% decreased significantly (2 (1–2) minutes vs 1 (1–2) minute; *p* < 0.05) (Table 4).

**Table 3.** Patient characteristics with ABCs

	Before training *N* = 29	After training *N* = 28	*p* value
Gestational age at birth (weeks), median (IQR)	27 + 6 (26 + 5–29)	27 + 2 (26–28 + 2)	0.19^a^
Birthweight (grams), median (IQR)	1016 (812–1199)	965 (692–1199)	0.51^a^
Male sex, no. (%)	22 (76)	16 (57)	0.14^b^
Cesarean delivery, no. (%)	13 (45)	15 (53)	0.51^b^
Singletons, no. (%)	22 (76)	22 (79)	0.57^b^
Apgar at 5 min, median (IQR)	8 (7–8)	7 (6–9)	0.25^a^
Days with respiratory support, no. median (IQR)	14 (8–32)	19 (9–31)	0.5^a^

^a^Independent samples Mann-Whitney U test

^b^Chi-square test

**Table 4.** ABC characteristics with FiO2-therapy

	Before training (ABC = 186)	After training (ABC = 168)	*p* value
ABC with SpO_2_ > 95%	73%	64%	ns^b^
Number of ABC, no. median (IQR)	4 (1–9)	4 (2–8)	0.64^a^
Depth of bradycardia, bpm median (IQR)	70 (60–75)	69 (61–75)	ns^a^
Duration of bradycardia, min median (IQR)	1 (1–1)	1 (1–1)	ns^a^
Depth of SpO_2_ < 80%, %	70 (62–76)	72 (61–77)	ns^a^
Duration SpO_2_ < 80%, min median (IQR)	2 (1–2)	1 (1–2)	0.03^a^
Baseline oxygen concentration, %	25 (21–31)	25 (21–30)	ns^a^
Max increase oxygen concentration, %	44 (39–52)	43 (37–51)	ns^a^
Duration of titration to baseline oxygen concentration, min median (IQR)	3 (2–16)	3 (2–7)	0.010^a^
Duration SpO_2_ > 95%, min median (IQR)	2 (0–7)	1 (1–3)	0.004^a^

^a^Independent samples Mann-Whitney U test

^b^Chi-square test

Although the baseline and the maximum increase of FiO_2_ during the ABC did not change, the duration of titrating oxygen back to the baseline concentration had a smaller range (3 (2–16) minutes to 3 (2–7) minutes; *p* < 0.05). There was no significant change in the occurrence of hyperoxaemia after ABCs (73% (135/186) vs 64% (108/168); ns), but the duration significantly decreased from 2 (0–7) minutes to 1(1–3) minute; *p* < 0.01 (Table 4).

## Discussion

We observed in this retrospective study that extra training and implementing a guideline in oxygen titration improved the compliance of caregivers in our NICU in oxygen targeting and a more prompt handling of ABCs. Preterm infants receiving oxygen spent significantly more time within the SpO_2_ target range of 85–95%, with a significant decrease in time SpO_2_ above 95%. The occurrence of hypoxaemia and hyperoxaemia during ABCs did not decrease, but both episodes lasted significantly shorter. This initiative in quality improvement had a positive effect, and if the observed reduction in the risk for hypoxaemia and hyperoxaemia could be maintained through repetitive training, it would be likely to improve the outcome of preterm infants.

Previous studies have reported a quality improvement in oxygen titration and oxygen saturation targeting, using an approach comparable to ours [6, 14, 19]. The problems were initially assessed, followed by embedding education and implementing a protocol, where after effectiveness was evaluated. In line with our findings, Ford et al. reported a significant improvement in time spent within the target range (90–95%) and a reduction of SpO_2_ above TR [14]. Lau et al. did not report the time spent within TR (85–92%) but observed a significant reduction in SpO_2_ ≥ 93% [19]. Also, in the study of Chow et al., the time spent within TR was not reported; they observed a decrease in severe ROP after introduction of an educational program combined with a titration protocol [6, 14, 19]. The fact that the findings were similar in most studies performed, including ours, makes it likely that this approach (training and guideline implementation) can be successful in most neonatal units.

Which part of the quality improvement that has contributed the most to the effect on the compliance of caregivers in oxygen titration and oxygen saturation targeting is unclear. Previous studies reporting the effect of guideline or education only were less successful compared to our study [2, 7, 11]. Clarke et al. reported no improved time within TR using a titration guideline. Arawiran et al. observed no improved adherence to TR (85–92%) after an education intervention with oral and online presentations, discussions of adverse effects of excessive oxygen, and displaying oxygen saturation distributions [2]. Also, Deuber et al. studied the effect of training with the aim to reduce hyperoxaemia and to increase caregivers’ knowledge. The time spent within TR (88–92%) was not reported; the time above TR was increased after training [11]. However, there are many variables that could have influenced the effect of training. Differences in content, approach and duration of the training but also the general workload, and the nurse to patient ratio could have influenced the results [3]. As part of our education, we discussed the results of our previous study, showing that SpO_2_ > 95% occurred in 79% of the ABCs where oxygen was increased [33]. During the training, we observed that caregivers felt personally addressed, resulting in behavioral change by better titration of oxygen during apnoeas.

It is clear that guidelines were not followed exactly, and compliance with the exact timing and step size was not measured. Nevertheless, when presented as part of the training, they provided a realistic framework on how to avoid hyperoxaemia, without increasing hypoxaemia. When the guideline was introduced and implemented in our unit, we took into account the factors that are important for adopting a guideline. Factors related to organization (i.e., support from physicians), to nurses (i.e., awareness of and attitudes to guidelines), to anticipated consequences (i.e., benefit to the patients and nurses’ work), and to the patient group (i.e., topic of the guideline) were identified as important factors for adopting a guideline [1]. To get all caregivers involved, the guideline was openly discussed during the training sessions.

Recently, we reported how nurses responded to ABC and handled the oxygen titration [33]. In a retrospective study in preterm infants on nCPAP, we observed that when extra oxygen was given to treat ABCs, iatrogenic hyperoxaemia occurred and lasted significantly longer than the bradycardia or hypoxaemia. Although the duration of hypoxaemia was comparable, the duration of hyperoxaemia was significantly longer (13 (4-30) minutes) in our previous study than to what we currently observed in the cohort before the intervention. A possible explanation could be the use of the “increase FiO_2_” key on the AVEA-ventilator. When this key is activated, the ventilator increases the oxygen concentration delivered to the infant for 2 min, where after the ventilator will return to prior settings. Nevertheless, training and guideline implementation significantly reduced the duration of hypoxaemia and hyperoxaemia even more. Apparently, nurses were more prompt in their handling when an ABC occurred, but also titrated more carefully. Poets et al. found an increased risk of adverse outcomes in preterm infants who experienced intermittent hypoxaemia, lasting for approximately 1 min or more [23]. This emphasizes the need for awareness and accurate handling of ABCs by the nurses.

In the recent years, there is an increasing interest in an automatically titration of oxygen in preterm infants. Closed-loop devices designed for monitoring and controlling the oxygenation in (ventilated) preterm infants are clinically used in research related context [8, 15, 30, 35]. These studies have shown that using automated oxygen control significantly increased time of %SpO_2_-wtr of approximately 8–24%, however, the time outside TR varied between studies. Most studies, but not all, reduced hyperoxaemia, and some also reduced hypoxaemia [8, 15, 30, 35]. Our study within the manual control showed comparable results with automatic devices concerning the increased time %SpO_2_-wtr and decreased time %SpO_2_ above TR. To make sure that this effect remains, repetitive training should be implemented in our unit.

Recent randomized controlled trials demonstrated a lower mortality in preterm infants when SpO_2_ was targeted 91–95% as compared to 85–89% [5, 24, 25, 27, 28]. In the time period, this observational study was performed; our local guidelines recommended 85–95% but were changed to 90–95% after the study. It is possible that this change could lead to different results when measuring the effect of training and guideline implementation. Jones et al. recently demonstrated that preterm infants with BPD were much more stable and less difficult to target when higher SpO_2_ targets were used [17].

A limitation is the retrospective character of our study. The training and oxygen titration guidelines were initiated for the quality improvement in our unit, and for this reason, the effect was audited by comparing before and after the interventions instead of a randomized trial. The dip in the frequencies of SpO_2_ 87–90% is associated with the generation of Masimo oximeters available in our unit at the time of this study, using an internal calibration algorithm that reduces the frequency of saturations of 87–90% and increases the frequency of higher values [16]. However, this would not have influenced the effect of training and guideline implementation as both groups were measured with the same oximeters.

Furthermore, we did not adjust for the contribution of the amount of ABCs of each patient, but we considered every ABC as an independent event because all ABCs are handled the same for each infant. An important factor that could have influenced the results is the workload of caregivers. However, the nurse to patient ratio, the number of patients, the severity of illness, and the NICU admission days were not different between the periods, which makes it unlikely that the workload differed between periods. In addition, based on the findings in recent large trials in oxygen saturation, in our unit, the TR was narrowed towards the higher end (90–95%). It is possible that not similar results will be reached as it will be more difficult to comply with a smaller TR.

## Conclusion

Based on the observations of this study, training of caregivers combined with an oxygen titration guideline, improved the compliance to stay within SpO_2_ target range in preterm infants. Also, the amount of hyperoxaemia reduced, without an increase of hypoxaemia. Thereby, oxygen was better titrated and reduced the duration of hyperoxaemia after ABCs.

## Abbreviations

ABC: Apnoea, bradycardia, cyanosis
BPD: Bronchopulmonary dysplasia
FiO2: Fraction of inspired oxygen
GA: Gestational age
LUMC: Leiden University Medical Center
nCPAP: Nasal continuous positive airway pressure
NICU: Neonatal intensive care unit
PDMS: Patient data management system
SpO_2_: Pulse oxygen saturation
TR: Target range
%SpO_2_-wtr: Proportion of time in percentage SpO_2_ was within the target range
